# Local Administration of AAV-BDNF to Subventricular Zone Induces Functional Recovery in Stroke Rats

**DOI:** 10.1371/journal.pone.0081750

**Published:** 2013-12-02

**Authors:** Seong-Jin Yu, Kuan-Yin Tseng, Hui Shen, Brandon K. Harvey, Mikko Airavaara, Yun Wang

**Affiliations:** 1 Center for Neuropsychiatric Research, National Health Research Institute, Zhunan, Taiwan; 2 Neural Protection and Regeneration Section, National Institute on Drug Abuse, Intramural Research Program, Baltimore, Maryland, United States of America; 3 Institute of Biotechnology, Viikki Biocenter, University of Helsinki, Helsinki, Finland; University of South Florida, United States of America

## Abstract

Migration of new neuroprogenitor cells (NPCs) from the subventricular zone (SVZ) plays an important role in neurorepair after injury. Previous studies have shown that brain derived neurotrophic factor (BDNF) enhances the migration of NPCs from SVZ explants in neonatal mice *in vitro*. The purpose of this study was to identify the role of BDNF in SVZ cells using AAV-BDNF in an animal model of stroke. BDNF protein production after AAV‐BDNF infection was verified in primary neuronal culture. AAV-BDNF or AAV-RFP was injected into the left SVZ region of adult rats at 14 days prior to right middle cerebral artery occlusion (MCAo). SVZ tissues were collected from the brain and placed in Metrigel cultures 1 day after MCAo. Treatment with AAV-BDNF significantly increased the migration of SVZ cells in the stroke brain in vitro. In another set of animals, AAV-GFP was co-injected with AAV-BDNF or AAV-RFP to label cells in left SVZ prior to right MCAo. Local administration of AAV-BDNF significantly enhanced recovery of locomotor function and migration of GFP-positive cells from the SVZ toward the lesioned hemisphere in stroke rats. Our data suggest that focal administration of AAV-BDNF to the SVZ increases behavioral recovery post stroke, possibly through the enhancement of migration of cells from SVZ in stroke animals. Regional manipulation of BDNF expression through AAV may be a novel approach for neurorepair in stroke brains.

## Introduction

Experimental data has supported the idea that brain derived neurotrophic factor (BDNF) has beneficial effects in animal models of stroke. For example, treatment with BDNF reduces the size of brain infarction when given prior to middle cerebral artery occlusion (MCAo) in rodents [1]. Similarly, intracerebroventricular administration of adeno-associated virus (AAV) carrying the BDNF gene reduces brain infarction [2]. Pretreatment with BDNF reduces ischemia -mediated changes in BAX [3]and TUNEL labeling [4], suggesting the protection from BDNF is mediated through the inhibition of apoptosis. 

De novo neurogenesis has been found in the subventricular zone (SVZ) of adult mammalian brain. Increasing apoptosis in SVZ cells was found in BDNF deficient mutant mice [5]. TrkB is one of the most prominent neurotrophin receptors, in truncated isoform, in the SVZ of non-stroke mice [6]. Activation of pan-neurotrophin receptor p75 by BDNF regulates the neurogenesis in adult SVZ [7]. In contrast, BDNF, delivered intraventricularly, did not increase the SVZ neurogenesis in non-lesioned rodents [5]. In the intact animal, neuroprogenitor cells (NPCs) originating in the SVZ mainly migrated to the olfactory bulb. The migration of NPCs from the SVZ toward olfactory bulb is enhanced by BDNF [8,9]. These data suggest that migration and/or neurogenesis of NPCs in SVZ can be modulated by BDNF in non-lesioned adult brain [10,11].

We have used a rat distal MCAo model to characterize time-dependent proliferation and migration of NPC in the SVZ [13]. In contrast to non-stroke animals, NPCs from the SVZ mainly migrate toward the lesioned brain region in stroke animals. Manipulation of the survival or migration of NPCs in SVZ alters behavioral function in the stroke animals [12,13]. The role of BDNF in NPCs after stroke is still not fully understood. Indirect evidence supports that BDNF has neuroreparative effects via SVZ in stroke animals. For example, intravenous administration of BDNF stimulated recruitment of NPCs migrate to the lesioned striatum after stroke [14]. Intraventricular infusion of BDNF antisense oligonucleotides blocked the expression of BDNF mRNA and attenuated skilled reaching recovery after stroke [15]. We recently demonstrated that repeated intranasal delivery of cocaine- and amphetamine-regulated transcript (CART) increased BDNF expression in SVZ and enhanced behavioral recovery after stroke [12]. CART enhanced the migration of SVZ explant cells, which was antagonized by BDNF blocking antibody [12]. Similarly, Chen et al reported that atorvastatin induced SVZ explant cell migration through BDNF upregulation and improved neurological function in stroke animals [16]. A recent study showed that bath application of BDNF promotes cell displacement in acute ischemic brain slices [17]. These indirect evidences support that BDNF may enhance the migration of SVZ cells in stroke brains. It is still not clear if increasing BDNF expression locally in SVZ enhances the cell migration and functional recovery in the ischemic brain.

In this study, we demonstrated a neuroregenerative effect of BDNF after stroke. We found that local overexpression of BDNF expression in the SVZ by AAV-BDNF enhanced migration of SVZ cells toward the lesioned hemisphere and induced recovery of locomotor function in stroke rats. 

## Materials and Methods

### Animals and MCAo

Adult male Sprague-Dawley rats, purchased from Charles River Laboratories Inc., were housed in an enriched environment by providing a toy (nylabone) or crinkle paper in their home cages with a 12 hour dark (6pm to 6 am) and 12 hour light (6 am to 6 pm) cycle. This study was carried out in strict accordance with the recommendations in the Guide for the Care and Use of Laboratory Animals of the National Institutes of Health. The protocol was approved by the Committee on the Ethics of Animal Experiments of the National Institutes of Health. All surgery was performed under anesthesia, and all efforts were made to minimize suffering. Rats were anesthetized with chloral hydrate (0.4 g/kg, i.p.). The right MCA was ligated with a 10-O suture and common carotids were clamped bilaterally by nontraumatic arterial clips to generate focal infarction in the cerebral cortex. The ligature and clips were removed after 90-min ischemia to allow reperfusion as previously described [12,13]. Core body temperature was maintained at 37 °C. The averaged size of lesion was 190-200 mm^3^ in the right cerebral cortex, as determined by T2WI as described previously [12,18]. No animal died during surgery or during post-stroke drug treatment. 

### AAV Production and *In vitro* characterization

The construction of dsAAV-GFP [19] and dsAAV-RFP [20] dsAAV-RFP packaging plasmids is are based on AAV serotype 2 genome. To generate dsAAV-BDNF, GFP was replaced with the human BDNF cDNA. Viral stocks were prepared using the triple-transfection method with modifications [21,22]. Briefly, twenty 15 cm dishes containing HEK293 cells at 85–95% confluency were transfected by the CaCl_2_ method with pHelper (Stratagene, La Jolla, CA), pdsAAV-GFP or pdsAAV-BDNF and pXR1 aka pXX12, a plasmid containing the rep/cap genes for serotype 1. Plasmids used for packaging AAV were generously provided by Dr. Xiao Xiao (UNC, Chapel Hill, NC). Approximately 48 hours post-transfection, cells were harvested, lysed by freeze/thaw, and purified by centrifugation on a CsCl gradient. Final samples were dialyzed in PBS, aliquoted and stored at −80°C until use. All vectors were titered by quantitative PCR using GFP CMV promoter as the target sequence.

Rat primary cortical neuron cultures were prepared as described previously [22] and transduced with 11 µl of AAV-BDNF in 1 ml media. Two days later, media was collected and 60 µl was analyzed for BDNF using an E_max_ ImmunoAssay Kit (Promega, Madison, WI). The secreted BDNF was calculated from a standard curve of recombinant human BDNF protein provide with kit. 

### Unilateral AAV injection into SVZ

For the *in vitro* metrigel study, AAV-BDNF or AAV-RFP (1 µl, 5x10^9^ viral genomes/µl) was stereotactically delivered at a speed of 0.5 µl/min into left SVZ (AP 0.12 mm, ML 2.1 mm, DV 3.5 mm to bregma). For the *in vivo* histological study, AAV-GFP (1 µl, 5x10^9^ viral genomes/µl) was co-administered with AAV-BDNF or AAV-RFP (1 µl, 5x10^9^ viral genomes/µl) to the left SVZ (total volume for each site = 2 µl). 

### Triphenyltetrazolium chloride (TTC) staining

AAV-BDNF or AAV-RFP was injected into the left SVZ region of adult rats at 14 days prior to right MCAo surgery. Two days after reperfusion rats were decapitated. The brains were removed and sliced into 2.0-mm-thick sections. The brain slices were incubated in a 2% TTC solution (Sigma, St. Louis) for 15 min at room temperature and then transferred into a 4% paraformaldehyde solution for fixation. The area of infarction in each slice was measured with a digital scanner and Imagetools programs (University of Texas Health Sciences Center). The volume of infarction in each animal was obtained from the product of average slice thickness (2 mm) and sum of infarction areas in all brain slices examined.

### Locomotor Behavior

Animals were individually placed in 42x42x31 cm behavioral chambers for 60 minutes (Accuscan activity monitor, Columbus, OH). The monitor contained 16 horizontal and 8 vertical infrared sensors spaced 2.5 cm apart. Locomotor activity was calculated using the number of beams broken by the animals after placement in the chamber. Four parameters were measured. 

Vertical activity (VACTV): The total number of beam interruptions that occurred in the vertical sensor. Horizontal activity (HACTV): the total number of beam interruptions that occurred in the horizontal sensor. Total distance travelled (TOTDIST): the distance traveled in centimeters. Movement time (MOVTIME): The amount of time, in seconds, the animals was in ambulation.

### Histology

Animals were anesthetized with chloral hydrate (400 mg/kg i.p.) and perfused transcardially with saline followed by 4% paraformaldehyde (PFA) in phosphate buffer (PB; 0.1 M; pH 7.2). The brains were dissected, post-fixed in PFA for 18 - 20 hours, and transferred to 18% sucrose in 0.1 M PB for at least 16 hours. Serial sections of the entire brain were cut at 25 µm thickness on a cryostat. Histological images were acquired using a QImaging Retiga EXi camera and Nikon 80i. GFP fluorescence was measured as previously described [12].

### SVZ explant culture

SVZ explant cultures were prepared from stroke rats. AAV-BDNF or AAV-RFP was injected into the SVZ region of adult rats at 14 days prior to MCAo surgery. SVZ tissues were collected 1 day after 90-min MCAo. SVZ explants were cultured within Matrigel (BD Biosciences) in Neurobasal medium containing 2% B27 supplement (Invitrogen). The distance of SVZ cell migration was examined from days 2-8 after culture [12]. 

### Statistical analysis

Values are means ± s.e.m. Unpaired t-test, 2-way ANOVA, and post-hoc Newman-Keuls test were used for statistical analysis. A statistically significant difference was defined as p < 0.05.

## Results

(A) AAV-BDNF infection enhances BDNF production in primary cortical neuronal culture.

**Figure 1:**
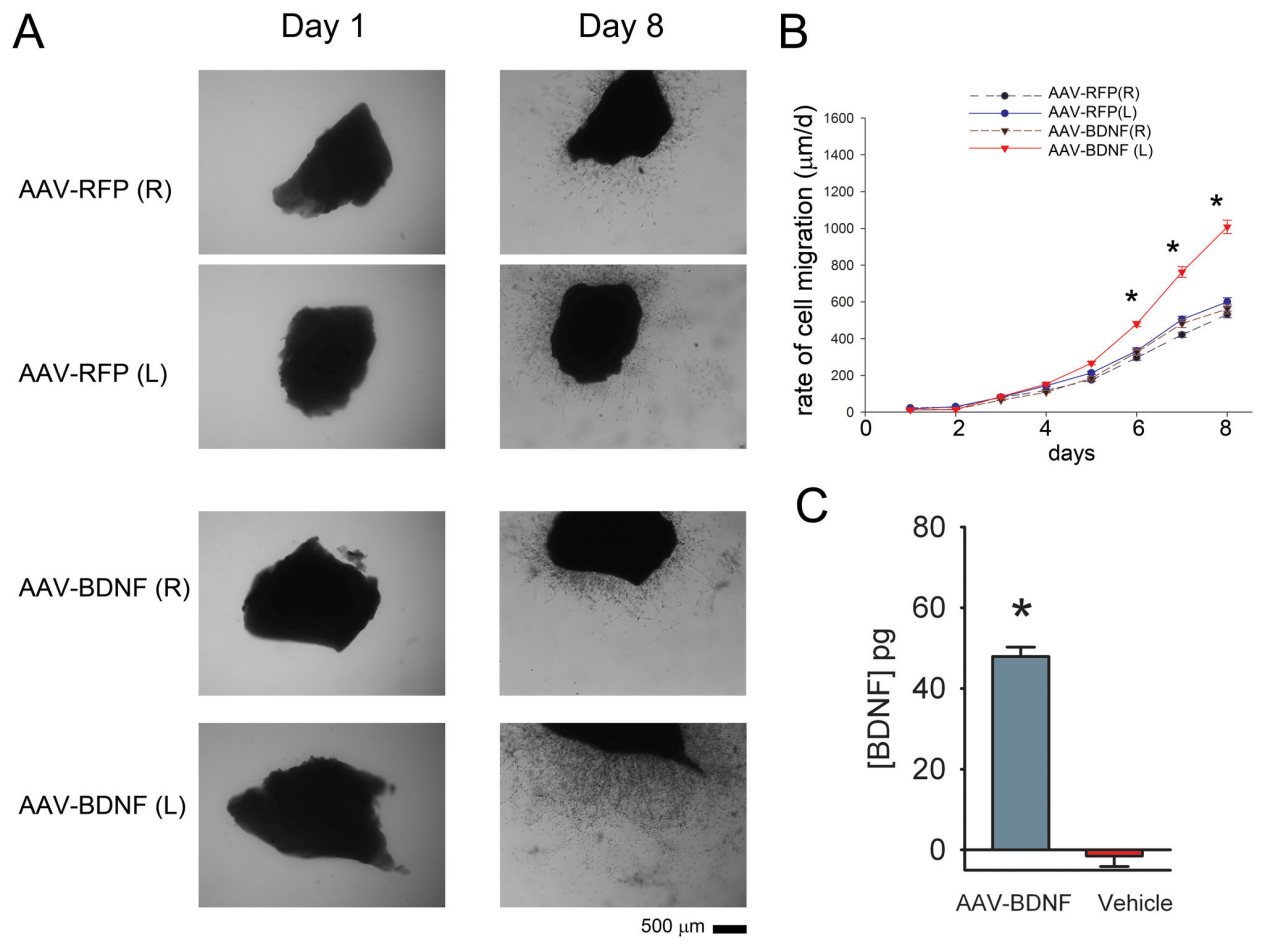
Treatment with AAV-BDNF enhances cell migration from SVZ explants derived from stroke brains. Adult rats were infected with AAV-BDNF or AAV-RFP unilaterally into the left (L) SVZ 2 weeks prior to right (R) MCAo. Bilateral SVZ tissues were harvested one day after MCAo and were cultured separately in Matrogel. The migration of SVZ cells from the explants was examined microscopically from days 1-8 after culture. (A) Representative images of SVZ explants from stroke animals pretreated with AAV-RFP (upper panels) or AAV-BDNF (lower panels). AAV-BDNF treatment increased cell migration from the left SVZ explants (AAV-BDNF-L) on DIV 8. There was minimal migration on DIV2. Calibration= 500 µm. (B)AAV-BDNF, compared to AAV-RFP, enhanced cell migration from the SVZ explants on DIV8. AAV-BDNF did not alter the migration of SVZ cells from the hemispheres contralateral to the viral injection (R). *p<0.001, 3- Way ANOVA + post-hoc Newman Keuls test. (C) AAV-BDNF increased BDNF secretion in primary cortical neurons. Rat primary cortical neurons (DIV6) were mock transduced or transduced with AAV-BDNF. Media was collected 2 days after transduction and analyzed for BDNF. Only cells transduced with AAV-BDNF showed significant increases above background (p<0.0001, t-test).

Rat primary cortical neurons were transduced with AAV-BDNF or vehicle. Media was collected for BDNF protein assay 2 days later. The production of BDNF protein was determined by ELISA. AAV-BDNF treatment significantly increased BDNF protein level in the media (Figure 1C, p<0.0001, t test). 

(B) AAV-BDNF infection enhances migration of SVZ explant cells from stroke rats.

Cell migration was examined from 144 SVZ explants *in vitro*. AAV-BDNF or AAV-RFP was administrated unilaterally into the left SVZ. Bilateral SVZ explants were harvested and then placed in Matrigel culture 1 day after right MCAo. The distance of SVZ cell migration from the explants was examined under the microscope from days 1-8 after culture. There was minimal migration on DIV2 (Fig 1A). Using a three way ANOVA, we found that AAV-BDNF significantly enhanced cell migration (F=146.450, p<0.001, Figure 1A, 1B). Post-hoc Newmann Keuls test analysis indicates that treatment with AAV-BDNF, compared to AAV-RFP, enhanced the distance of cell migration from the Left SVZ on DIV 6, 7, and 8 (p<0.001, Figure 1B). These data suggest that local AAV-BDNF infection enhances migration of cells from SVZ explants *in vitro*. 

(C) Pre-stroke administration of AAV-BDNF to the contralateral SVZ did not alter the size of infarction.

AAV-BDNF or AAV-RFP was administered locally to the left SVZ at 14 days prior to the right MCAo in 10 rats. Cerebral infarction was examined by TTC staining 2 days after MCAo. We found that pretreatment with AAV-BDNF did not significantly alter the size of infarction (AAV-BDNF 128.0 ± 26.9 mm^3^ vs AAV-RFP: 112.4 ± 18.3 mm^3^, p=0.645, t test).

(D) Behavioral recovery induced by AAV-BDNF treatment in stroke rats.

**Figure 2:**
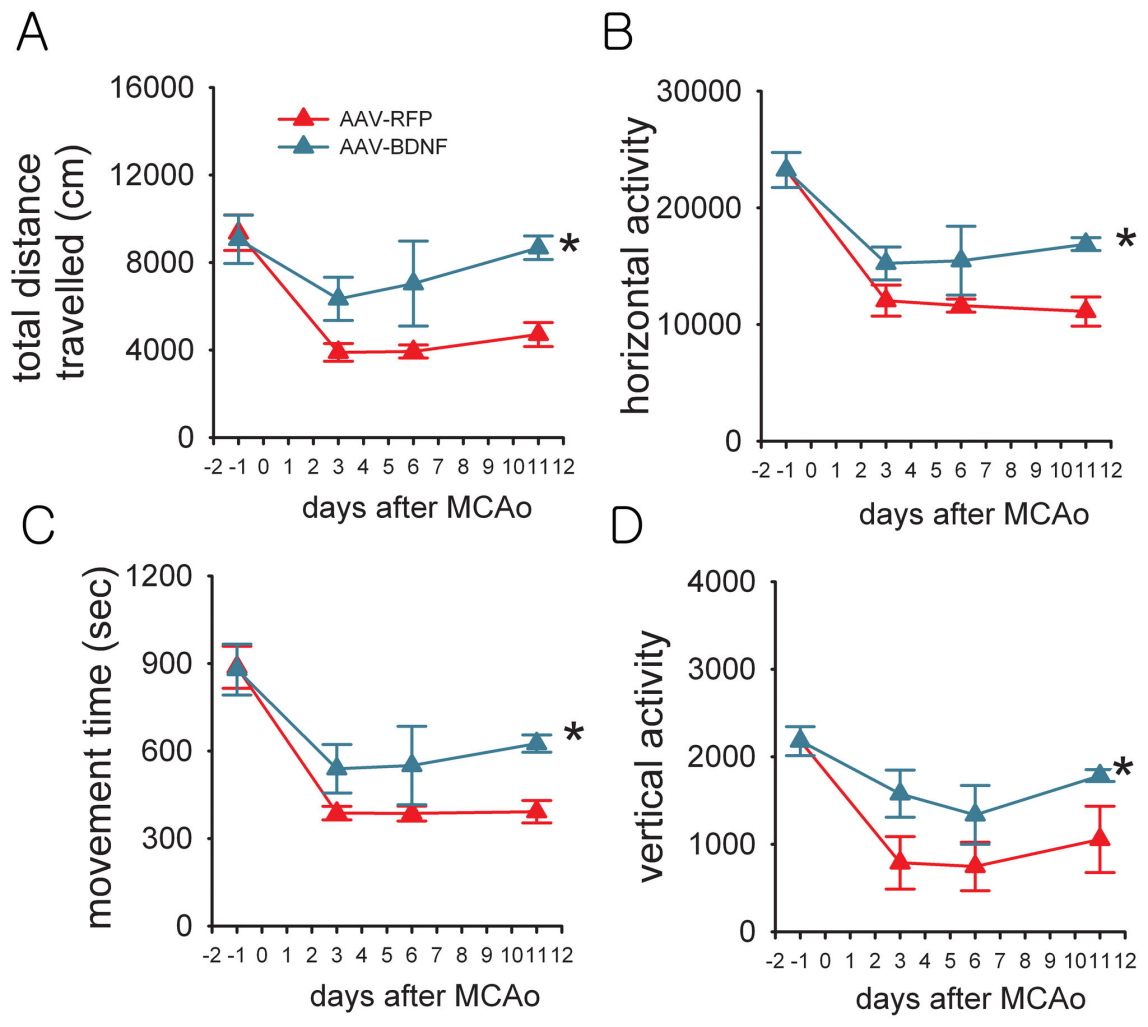
Local treatment with AAV-BDNF improved locomotor behavior in stroke rats. (A-D) Abscissa indicates that rats were subjected to a 90- min MCAo surgery on day 0. AAV-RFP or AAV-BDNF was administered locally to left SVZ to animals 14 days prior to MCAo. Animals were placed individually into the activity chamber to monitor 4-hour locomotor behavior. Behavioral measurements were carried out in automated activity chambers for 4 hours at 1 day before, 3, 6, and 11 days after MCAo. No difference was found before MCAo on day -1. Rats that received AAV-BDNF treatment show enhanced recovery in motor function demonstrated by significant increases in (A) total distance traveled, (B) horizontal activity, (C) movement time, and (D) vertical activity compared to the control stroke group receiving AAV-RFP (p<0.05, 2-way ANOVA; * p<0.05, post-hoc Newman-Keuls test).

AAV-BDNF or AAV-RFP was given locally to the left SVZ 14 days prior to MCAo in 16 rats. Animals were placed individually into the activity chamber to monitor 4-hour horizontal activity, total distance travelled, movement time and vertical activity 1-day prior to and 3, 6, and 11 days after MCAo or sham surgery. Administration of AAV-BDNF did not alter locomotor activity prior to MCAo (Figure 2) or after sham surgery (data not shown). Animals receiving AAV-BDNF demonstrated behavioral improvement post-stroke. Horizontal activity, total distance travelled, movement time, and vertical activity were significantly increased after MCAo in AAV-BDNF, compared to AAV-RFP, -treated rats (Figure 2, p<0.05, 2-way ANOVA; p<0.05, post-hoc Newman-Keuls test). These results demonstrate that focal administration of AAV-BDNF in non-lesioned side SVZ is effective in improving the functional outcome in stroke rats. 

(E) AAV-BDNF increases migration of SVZ cells toward the lesioned side hemisphere in the stroke rats. 

**Figure 3:**
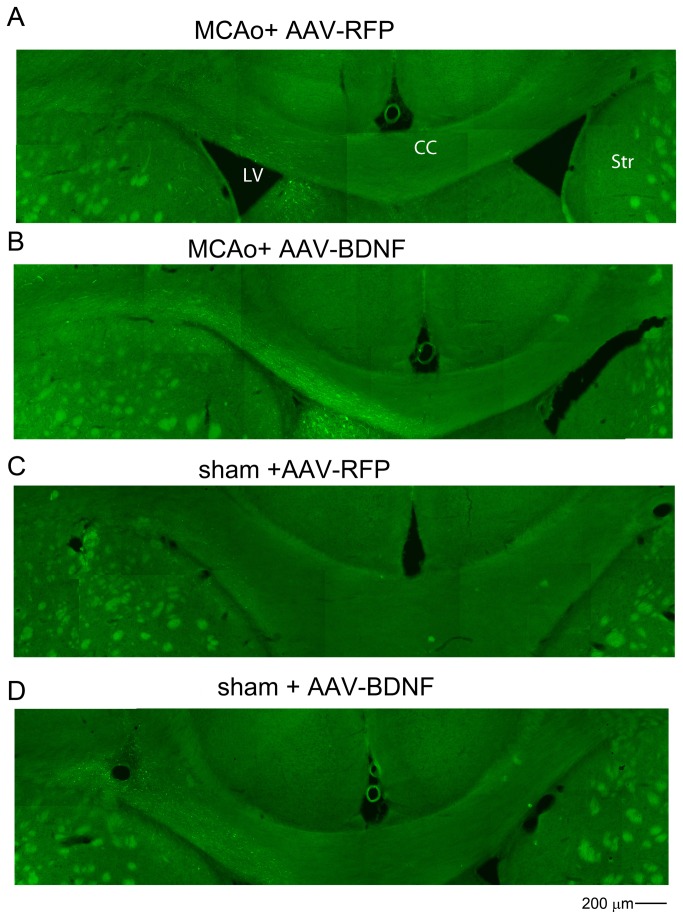
Increases in BDNF expression in SVZ enhance cell migration in stroke rats. Local administration of AAV-BDNF in the Left SVZ enhanced cell migration from SVZ toward the ischemic cortex (Right) in stroke animals. (A-D) Animals were treated with AAV-BDNF/AAV-GFP or AAV-RFP/AAV-GFP in the contralateral (Left) SVZ 14 days prior to (A,B) MCAo or (C,D) sham surgery. Pretreatment with (B) AAV-BDNF, compared to (A) AAV-RFP, increased migration of GFP(+) cells from SVZ, along the corpus callosum, toward lesioned side hemisphere (Right) on day 12 post-stroke. (C,D) Limited cell migration was found in the corpus callosum of non-stroke rats. CC: corpus callosum; LV: Left ventricle; Str: striatum .

**Figure 4:**
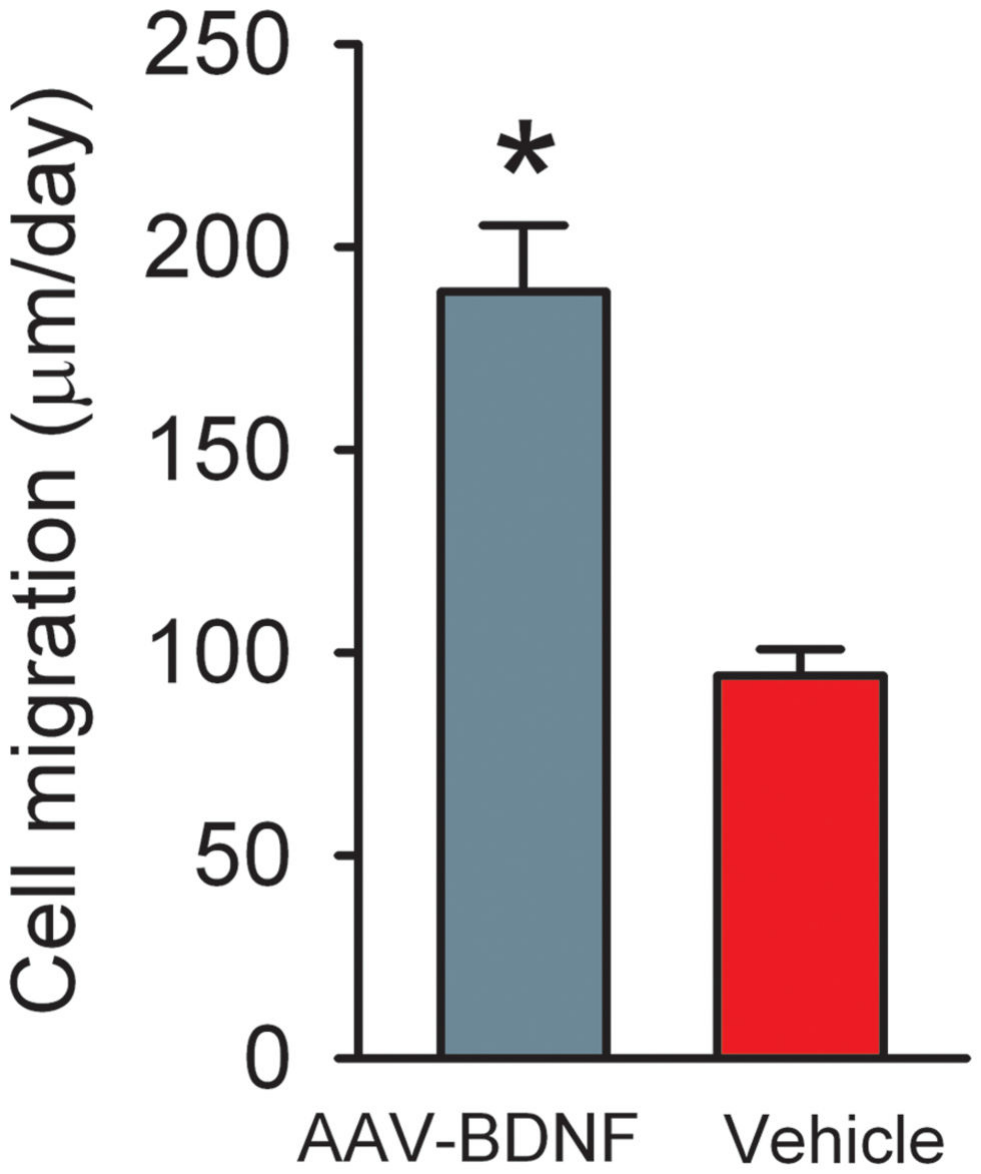
Rate of cell migration was significantly increased in stroke animals treated with AAV-BDNF. The rate of cell migration (µm per day) was calculated by averaging the distance of GFP (+) cells travelling from the Left SVZ toward the ischemic hemisphere (Right) after right MCAo treatment in 12 days. *p<0.05, t test.

A total of 16 rats were treated with AAV-BDNF or AAV-RFP in the non-lesioned side (Left) SVZ at 7 days prior to MCAo or sham surgery. SVZ cells were also labeled with GFP by co-administration of AAV-GFP. Brain tissues were harvested 12 days after MCAo. GFP(+) cells migrated bilaterally from the injection site, but mainly toward to the lesioned hemisphere in stroke animals (Figure 3). Limited cell migration along the corpus callosum was found in non-stroke animals. Treatment with AAV-BDNF enhanced GFP(+) cell migration from the contralateral SVZ toward the ischemic cortex only in stroke animals (Figure 3). The rate of cell migration (µm per day) was calculated by averaging the distance of GFP (+) cells travelling from the contralateral SVZ toward ischemic hemisphere after MCAo treatment (i.e. 12 days). AAV-BDNF infection significantly increased the rate of cell migration in stroke animals (Figure 4, p=0.011, t test).

## Discussion

Overexpression of BDNF via AAV has been shown to induce neural protection and regeneration in various lesion models. AAV-BDNF infection increased the neural and progenitor cell survival in striatum in the quinolinic acid rodent model of Huntington’s disease [23,24]. Pretreatment with AAV-BDNF, given i.c.v., reduced the size of brain infarction in the focal ischemic model [2]. In this study, AAV-BDNF was administered locally to the non-lesioned side SVZ, a region that is actively involved in neural repair after stroke. We demonstrated that AAV-BDNF delivered to the SVZ contralateral to MCAo did not alter the size of infarction, however, enhanced endogenous NPC migration from SVZ and improved functional recovery in stroke animals.

The migration of endogenous NPCs from SVZ of stroke rats was examined in culture and *in vivo*. Using the *in vitro* Metrigel culture technique, we demonstrated an enhanced cell migration from SVZ explants receiving AAV-BDNF. There was no change in migration from the SVZ contralateral to viral injection, suggesting that the trophic response is limited to the site of infection. In the *in vivo* study, we administered AAV-BDNF to the SVZ region, contralateral to the MCAo. We found that migration of labeled SVZ cells in the corpus callosum towards the ischemic hemisphere was significantly enhanced by AAV-BDNF in stroke animals. In contrast, there was limited cell migration in the corpus callosum in non-stroke rats, treated with either AAV-BDNF or AAV-RFP. We also measured horizontal and vertical activity in stroke rats. A significant recovery of behavioral function was found in animals receiving AAV-BDNF. These data suggest that local injection of AAV-BDNF facilitates the migration of SVZ cells to the contralateral hemisphere in stroke rats. 

BDNF has been shown to enhance migration of NPCs in SVZ in non-lesioned animals; the migration is mainly toward the olfactory bulbs [9–11]. Limited reports indirectly support the effect of BDNF on SVZ cell migration in stroke animals. For example, systemic administration of BDNF enhanced recruitment of NPCs into the ipsilateral striatum after stroke [14]. Administration of drugs, such as CART or atorvastatin, increased the expression of BDNF and enhanced migration of SVZ cells [12,25]. However, in these studies, systemically applied BDNF or its agonists can interact with multiple anatomical sites. It is not clear if the improvement of cell migration is mediated through an action in SVZ or the target lesioned sites [1]. In current study, overexpression of BDNF was achieved locally in the SVZ via recombinant AAV delivery. Our data support a trophic response with BDNF in the SVZ to enhance the migration of these cells toward the lesioned target. 

Cerebral ischemia can activate proliferation of NPCs for endogenous repair. However, most of these cells die and do not migrate to the lesioned area [13]. We previously demonstrated that enhancing survival of the endogenous neural progenitor cells (NPCs) in SVZ by a p53 inhibitor, pifithrin-α, improved functional recovery in stroke animals [13]. In this study, we demonstrated local AAV-BDNF administration enhanced the migration of the SVZ cells in stroke rats. Future studies will examine the additive/ synergisitic effect of combined treatment for enhancing the survival and migration in stroke animals. 

A recent report indicated that activation of AMPA receptors post-stroke enhances behavioral recovery through BDNF signaling in stroke mice [26]. Similarly, systemic administration of the N-methyl-D-aspartate (NMDA) receptor blocker MK-801 suppressed neurogenesis in hippocampus after MCAo in rats [27]. On the other hand, local overexpression of glutamate transport-1 (GLU1) in the ischemic cortex reduced brain damages in stroke rats [28]. These data support two opposite reactions of excitatory amino acids in stroke. Activation of glutamate receptors enhances neurodegeneration in the ischemic region, while it induces neuroregeneration through BDNF in non-injured regions. These conflicting responses may partially underlie the failure of using GluR antagonists in clinical trials in stroke patients [29]. A better approach would be reducing glutamate concentration locally in the lesioned area, for example targeted overexpression of GLT-1 in the ischemic brain region as we previously described [28], and boosted endogenous neurorepair in non-lesioned region by enhancing the BDNF expression at the SVZ as presented in this study. 

## Conclusion

We demonstrated that focal delivery of AAV-BDNF to the non-lesioned side SVZ increased migration of NPCs in SVZ and improved behavioral function in stroke animals. Our results may provide a new treatment target at the non-lesioned side SVZ for stroke patients through gene or recombinant protein therapy. 
